# LncRNA BE503655 inhibits osteosarcoma cell proliferation, invasion/migration via Wnt/β-catenin pathway

**DOI:** 10.1042/BSR20182200

**Published:** 2019-07-29

**Authors:** Qiang Huang, Shu-yan Shi, Hai-bo Ji, Shu-xing Xing

**Affiliations:** 1 Department of Orthopedic Surgery, Chengdu Fifth People’s Hospital, Chengdu, Sichuan Province, China; 2 Department of Pathology, Chengdu Fifth People’s Hospital, Chengdu, Sichuan Province, China

**Keywords:** lncRNA BE503655, Osteosarcoma, Wnt/β-catenin

## Abstract

**Aim:** In previous studies, numerous dysregulated long non-coding RNAs (lncRNAs) were identified by RNA-sequencing (RNA-seq). However, the relationship between lncRNA and osteosarcoma remains unclear. In the present study, the function and mechanism of lncRNA BE503655 were investigated. **Methods:** Transwell, cell cycle and proliferation were used to evaluate the function of lncRNA BE503655. Real-time PCR and Western blotting were used to detect the expression of lncRNA BE503655 and β-catenin. **Results:** LncRNA BE503655 is overexpressed in human osteosarcoma and osteosarcoma cell lines. Knockdown lncRNA BE503655 suppresses cell proliferation, invasion and migration. High expression of BE503655 was significantly related to Enneking stage, distant metastasis and histological grade. Moreover, we also provided evidences that lncRNA BE503655 played its functions dependent on regulation of Wnt/β-catenin signaling in osteosarcoma. **Conclusion:** Taken together, we verified the role of lncRNA BE503655 and provided possible mechanism in osteosarcoma. Our study provided new insights into clinical treatment of osteosarcoma and further intervention target.

## Introduction

Osteosarcoma is the most prevalent primary malignant bone cancer and is most common in children and teenagers [1]. Approximately 20% of patients have metastasis when they are diagnosed [2]. It is quite common that people often die due to distant metastasis [3]. The metastasis of tumor is involved in various processes, including invasion [4], angiogenesis [5] and epithelial–mesenchymal transition (EMT) process [6]. Despite remarkable progress in clinical treatment, such as neoadjuvant chemotherapy [7] and surgery, has greatly improved the patients’ survival rates, there still exists numerous patients suffering from distant metastasis. Thus, exploring new targets for osteosarcoma are clearly warranted.

Long non-coding RNA (lncRNA) is a kind of non-coding RNA longer than 200 nucleotides in length with limited coding potential [8]. Many studies have revealed the role of lncRNAs involved in different biological activities including X-chromosome silence [9], chromatin modification [10], transcriptional activity [11]. For example, lncRNA FILNC1 represses c-Myc-mediated energy metabolism and inhibits renal tumor development [12]. lncRNA TSLNC8 is a tumor suppressor that inactivates the IL-6/signal transducer and activator of transcription (STAT) 3 (STAT3) signaling pathway [13]. Importantly, accumulating evidences have revealed that aberrant expression of lncRNA will lead to various human cancers [14,15]. Although large number of studies have demonstrated the function of lncRNA in different diseases, there is no doubt that exploring more and more lncRNAs will provide new choice for drug administration in clinical treatment.

In previous studies, numerous dysregulated lncRNAs were identified by RNA-sequencing (RNA-seq) [16]. However, the relationship between lncRNA and osteosarcoma remains unclear. In the present study, the function and mechanism of lncRNA BE503655 was investigated. LncRNA BE503655 is overexpressed in human osteosarcoma and osteosarcoma cell lines. Knockdown lncRNA BE503655 suppresses cell proliferation and invasion. High expression of BE503655 was significantly related to Enneking stage, distant metastasis and histological grade. Moreover, we also provided evidences that lncRNA BE503655 played its functions dependent on regulation of Wnt/β-catenin signaling in osteosarcoma. Taken together, we verified the role of lncRNA BE503655 and provided possible mechanism in osteosarcoma. Our study provided new insights into clinical treatment of osteosarcoma and further intervention target.

## Materials and methods

### Patients’ tissues collection

Osteosarcoma tissues and adjacent tissues were collected from 61 patients. None of the patients received chemotherapy or radiotherapy before sample collection. All the studies were approved by the Ethical Committee of West China Hospital and all patients gave informed consent.

### Cell culture and transfection

The cell lines, MG63 and HOS used in the present study were purchased from American Type Culture Collection (ATCC). MG63 and HOS cells were cultured in DMEM (Gibco, U.S.A.) containing 10% FBS (Gibco, U.S.A.). Cells were maintained at 37°C in a humidified atmosphere with 5% CO_2_.

The siRNAs were purchased from GenePharma (Shanghai, China). The knockdown efficiency was verified with MG63 cells using quantitative real-time polymerase chain reaction (qRT-PCR). The overexpression vector of β-catenin was purchased from GenePharma (Shanghai, China). Transfections were performed using Lipofectamine 2000 according to the manufacturer’s instructions (Invitrogen, CA, U.S.A.).

### Cell proliferation

MG63 cells were used to detect the cell proliferation rates. CCK8 assay kit (Dojindo, Japan) was used to measure the proliferation rates. Cells were seeded (1000 cells per well) into 96-well plates. The results were collected every 24 h until 96 h.

### Cell cycle

MG63 cells deprived of FBS were starved for 6 h before recovery in complete medium for another 24 h. The cells were fixed in 50% ethanol for cell cycle analysis. All the samples (20000 events collected per sample) were analyzed by flow cytometry.

### Transwell assay

MG63 cells were re-suspended in 100 μl serum-free medium and were plated in the top chamber of each insert (8-μm pore size, Corning, U.S.A.) with a Matrigel-coated membrane (BD Bioscience, San Jose, U.S.A.) for the transwell assay. Lower chambers of the inserts were filled with DMEM with 10% FBS. Twenty-four hours later, cells invaded/migrated to the lower surface of the insert were fixed, stained and counted under a light microscope.

### Real-time PCR

Total RNA from the serum samples and cultured MG63 cells were isolated with TRIzol Reagent (Thermo Fisher Scientific, MA, U.S.A.). Quantitative analysis of qRT-PCR was carried out using SYBR Premix ExTaq™ (TaKaRa, Dalian, China). Relative expression levels of lncRNA BE503655 and β-catenin mRNA were calculated by using the 2^−ΔΔ*C*^_T_ method.

### Western blotting

Proteins from MG63 cells were extracted by using the RIPA buffer. BCA assay was utilized to measure the protein concentration. The extracted proteins were then subjected to 10% SDS/PAGE electrophoresis and transferred into the PVDF membranes. The membranes were blocked with 5% non-fat milk and incubated with anti-β-catenin (Santa Cruz, CA, 1:1000), anti-c-myc (Santa Cruz, CA, 1:1000), anti-cyclin D (Santa Cruz, CA, 1:1000), anti-MMP2 (Santa Cruz, CA, 1:1000), anti-GAPDH (Santa Cruz, CA, 1:1000), overnight at 4°C. After washing five times (6 min each time), membranes were incubated with HRP–conjugated secondary antibodies (Abcam, CA, U.S.A.) for 1 h at room temperature. The bands were visualized by using the ECL kit (Thermo Fisher Scientific, Rockford, U.S.A.).

### Nuclear and chromatin RNA fraction

Nuclear and cytoplasmic fractions of MG63 cells were partitioned using a Protein And RNA Isolation System (PARIS) Kit (Thermo Fisher Scientific). A total of 10^7^ fresh cultured cells were collected, placed on ice and resuspended with 500 μl ice-cold cell fractionation buffer. Cells were incubated on ice for 10 min. Samples were centrifuged at 500×***g*** for 5 min, and then the cytoplasmic fraction was carefully aspirated away from the nuclear pellets. RNA isolation from chromatin and nucleoplasm was performed as described previously.

### Statistical analysis

All the graphs plotting and data analysis were performed by using the SPSS software 21.0. All the data were shown as mean ± standard deviation. The significant differences between different groups were analyzed by *t* test (comparison for two groups) or χ^2^ test or one-way ANOVA (comparison for more than two groups). *P*<0.05 was considered to be statistically significant.

## Results

### LncRNA BE503655 is overexpressed in human osteosarcoma and osteosarcoma cell lines

**Figure 1:**
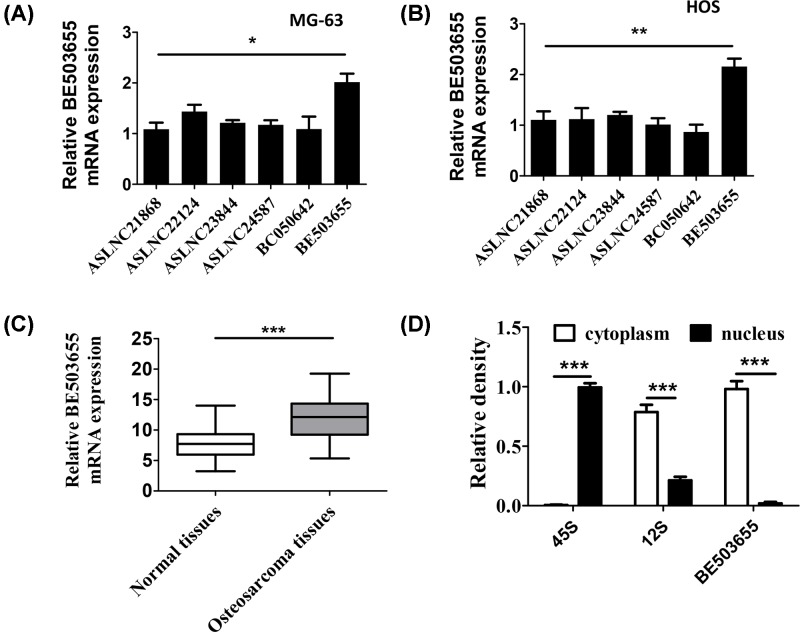
BE503655 is up-regulated in human osteosarcoma and osteosarcoma cell lines (**A,B**) RT-PCR analysis was performed to examine the expression of lncRNAs in MG-63 and HOS cell lines. (**C**) RT-PCR analysis was used to determine the expression of BE503655 in adjacent normal tissues and osteosarcoma tissues. (**D**) Nuclear separation experiment results showed that BE503655 mainly exists in the cytoplasm. All data were expressed as the mean **±** SD (*n*=3). *P<0.05, **P<0.01, ***P<0.001.

We performed RT-PCR analysis to examine the expression of these up-regulated lncRNAs (ASLNC21868, ASLNC22124, ASLNC23844, ASLNC24587, BE503655 and BC050642) in MG-63 and HOS osteosarcoma cell lines to investigate the potential function in osteosarcoma. As shown in Figure 1A,B, among all these up-regulated lncRNAs in tumor tissues, BE503655 expression was the most up-regulated in MG-63 and HOS cell lines. In addition, consistent with previous studies, we confirmed that the expression of BE503655 in osteosarcoma tissues is significantly up-regulated (Figure 1C). Moreover, nuclear separation experiment was conducted to confirm the location of BE503655 in HOS cells, we found that BE503655 is mainly expressed in the cytoplasm (Figure 1D).

### LncRNA BE503655 expression is associated with osteosarcoma patients’ clinical progression

To investigate the clinical significance of BE503655 expression in osteosarcoma patients, total 61 osteosarcoma tissue samples were extracted and examined for their mRNA levels. Then, the relationship between BE503655 expression and pathological features of osteosarcoma was analyzed. All samples’ mRNA levels were divided into ‘high-expression’ (*n*=34) and ‘low-expression’ (*n*=27) BE503655 groups according to the median value of BE503655 expression. As shown in Table 1, high expression of BE503655 was significantly related to Enneking stage (*P*=0.019), distant metastasis (*P*=0.013) and histological grade (*P*=0.034). However, there was no significant correlation between BE503655 expression and age, gender and tumor size.

**Table 1:** Association between BE503655 expression and clinicopathological characteristics in 61 osteosarcoma patients

Variable	LncRNA BE503655 expression		χ^2^	*P*-value
	Low expression (*n*=27)	High expression (*n*=34)		
Age (years)			0.041	0.839
<18	12	16		
≥18	15	18		
Gender			0.394	0.530
Male	18	20		
Female	9	14		
Enneking stage			5.522	**0.019**
I–IIA	20	15		
IIB–III	7	19		
Size (8 cm)			0.339	0.560
<8	23	27		
≥8	4	7		
Distant metastasis			6.231	**0.013**
Absent	19	13		
Present	8	21		
Histological grade			4.475	**0.034**
G1–G2	22	19		
G3–G4	5	15		

Significant difference between the two groups was considered as *P*<0.05 and is indicated in bold.

### LncRNA BE503655 knockdown suppresses cell proliferation, invasion/migration in osteosarcoma cells

**Figure 2:**
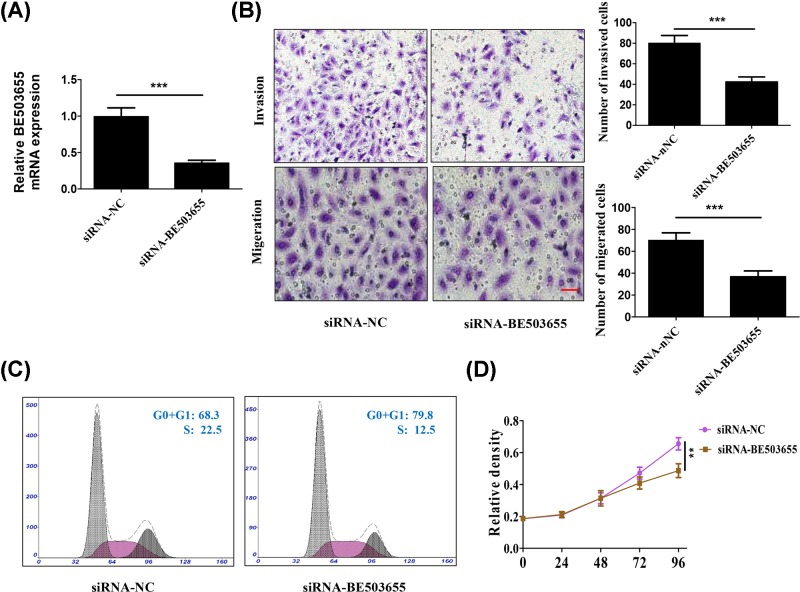
BE503655 knockdown suppresses proliferation, invasion in osteosarcoma cells (**A**) RT-PCR analysis was used to determine the mRNA expression of BE503655 in MG-63 cells. (**B**) Transwell assay was used to determine the role of BE503655 in the invasion and migration of MG-63 cells. Scale bar = 100 μm. (**C**) Flow cytometry was performed to detection the cell cycle of osteosarcoma cells. (**D**) CCK8 assay was used to investigate the role of BE503655 in the proliferation of MG-63 cells. All data were expressed as the mean **±** SD (*n*=3). **P<0.01, ***P<0.001.

To further determine the role of BE503655 in osteosarcoma, we knocked down BE503655 with siRNAs in HOS cells (Figure 2A). Transwell analysis results demonstrated that BE503655 silencing obviously reduced osteosarcoma cell invasion and migration (Figure 2B). Cell cycle detection showed that BE503655 down-regulation markedly inhibited the percent of osteosarcoma cells in S phase (Figure 2C). In addition, BE503655 silencing markedly inhibited the osteosarcoma cell proliferation by CCK8 assay (Figure 2D).

### BE503655 regulates Wnt/β-catenin pathway in osteosarcoma cells

**Figure 3:**
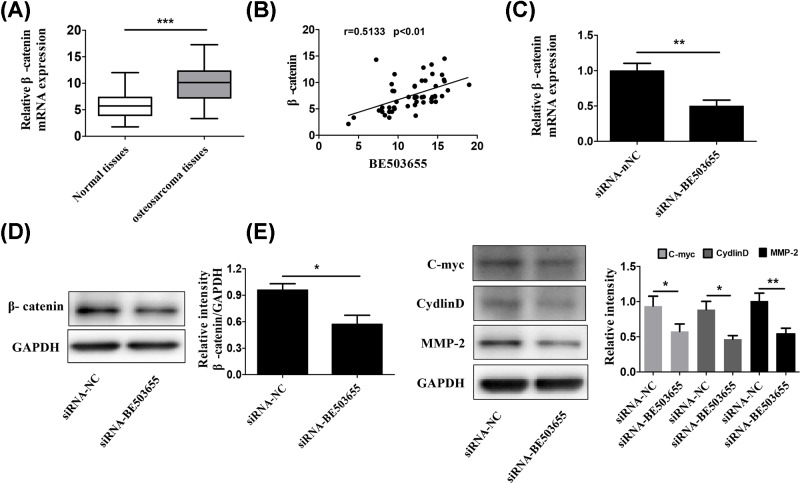
BE503655 regulates Wnt/β-catenin pathway in osteosarcoma cells (**A**) RT-PCR analysis was used to determine the mRNA expression of β-catenin in adjacent normal tissues and osteosarcoma tissues. (**B**) Correlation analysis showed that the expression of β-catenin was positively correlated with BE503655 expression. (**C**) RT-PCR analysis was used to determine the mRNA expression of β-catenin in MG-63 cells. (**D**) Western blotting analysis was used to examine the protein expression of β-catenin. (**E**) The target genes of Wnt/β-catenin pathway (C-myc, cyclin D and MMP-2) were detected by Western blotting analysis. All data were expressed as the mean **±** SD (*n*=3). *P<0.5, **P<0.01, ***P<0.001.

To explore BE503655-mediated potential molecular mechanism, we performed QT-PCR to screen signaling pathways, including mTOR, JNK and Wnt/β-catenin pathways, were confirmed to be mediated in human cancers (data were not shown). We analyzed the association of BE503655 with cancer-related signaling pathways and found that β-catenin expression was up-regulated in osteosarcoma tissues (Figure 3A), and was positively correlated with BE503655 expression (Figure 3B). When knocking down BE503655 in HOS cells, β-catenin mRNA level (Figure 3C) and protein expression (Figure 3D) were all significantly decreased. Furthermore, we found that some target genes of Wnt/β-catenin pathway were also down-regulated in siRNA-BE503655 silenced HOS cells (Figure 3E). These data indicated that BE503655 regulates Wnt/β-catenin signaling in osteosarcoma cells.

### LncRNA BE503655 knockdown inhibits osteosarcoma cell proliferation, invasion via wnt/β-catenin signaling

**Figure 4:**
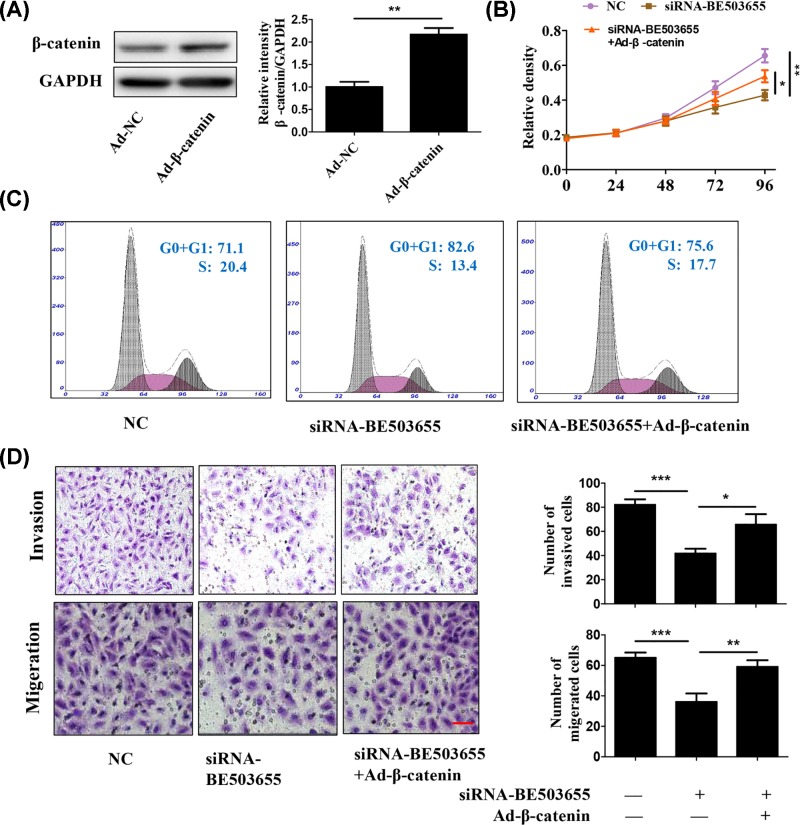
BE503655 knockdown inhibits osteosarcoma cell proliferation, invasion via wnt/β-catenin signaling (**A**) Western blot analysis was used to determine the protein expression of β-catenin, in osteosarcoma cell line. (**B**) CCK8 assay was used to investigate the role of β-catenin on the effect of BE503655 in the proliferation of MG-63 cells. (**C**) Flow cytometry results showed that the percent of osteosarcoma cells in S phase inhibited by BE503655 down-regulation was blocked after β-catenin overexpression. (**D**) Transwell assay was used to determine the role of β-catenin on the effect of BE503655 in the invasion and migeration of MG-63 cells. Scale bar = 100 μm. All data were expressed as the mean **±** SD (*n*=3). Each value of *P<0.05,**P<0.01, ***P<0.001 was deemed to have significant differences.

Given that BE503655 expression was positively correlated with Wnt/β-catenin pathway, we wonder whether BE503655 plays its function via regulated the Wnt/β-catenin pathway. The β-catenin expression was overexpressed by adenovirus (Figure 4A). CCK8 assay results showed that BE503655 silencing inhibited osteosarcoma cell proliferation was partially reversed by overexpression of β-catenin (Figure 4B). In addition, the percent of osteosarcoma cells in S phase inhibited by BE503655 down-regulation was blocked after β-catenin overexpression (Figure 4C). Consistent with the above results, transwell assay showed that BE503655 silencing obviously reduced osteosarcoma cell invasion and migration, but was partially blocked by up-regulation of β-catenin (Figure 4D). Taken together, above results suggested that lncRNA BE503655 played its functions dependent on regulation of Wnt/β-catenin signaling in osteosarcoma cells.

## Discussion

In previous studies, numerous dysregulated lncRNAs were identified by RNA-seq [16]. However, the relationship between lncRNA and osteosarcoma remains unclear. In the present study, the function and mechanism of lncRNA BE503655 were investigated. LncRNA BE503655 is overexpressed in human osteosarcoma and osteosarcoma cell lines. Knockdown lncRNA BE503655 suppresses cell proliferation and invasion. High expression of BE503655 was significantly related to Enneking stage, distant metastasis and histological grade. Moreover, we also provided evidences that lncRNA BE503655 played its functions dependent on regulation of Wnt/β-catenin signaling in osteosarcoma. Taken together, we verified the role of lncRNA BE503655 and provided possible mechanism in osteosarcoma. Our study provided new insights into clinical treatment of osteosarcoma and further intervention target.

LncRNAs, as long-stranded non-coding RNA with long sequence and complex spatial structure [17], have more information content than other non-coding RNAs. The mechanisms of lncRNA are also diverse, including *cis*-mechanism or *trans*-mechanism [18], and regulate histidine-modified recruitment [19]. Studies in the past few years have revealed the role of lncRNA in the cardiovascular disease [20], gastric cancer [10], colorectal cancer [21] and diabetes [22]. To date, limited studies demonstrated the role of lncRNA in osteosarcoma. This is the first study on lncRNA BE503655 in osteosarcoma and we found that lncRNA BE503655 can inhibit cell proliferation and invasion though regulating Wnt/β-catenin signaling pathway.

Previous studies have shown that lncRNA BE503655 was up-regulated in osteosarcoma tissues. In our study, qRT-PCR was utilized to detect the expression of lncRNA BE503655 in osteosarcoma cell lines and tumor tissues compared with normal tissues, and we found that the expression of lncRNA BE503655 in osteosarcoma cell lines and tissues was significantly higher than the control group. It is well known that proliferation and invasion are all essential manifestations of tumor development. It is always a central point to reduce the proliferation and inhibit the invasion of tumor cells. We found that knockdown of lncRNA BE503655 suppresses cell proliferation and invasion. Although we proved the function of lncRNA BE503655 in osteosarcoma, there still exists problems that need to be solved. Further studies are needed to delineate the targeting molecular of lncRNA BE503655, including elucidation of function of the molecular binding proteins.

In summary, our study verified the function and mechanism of lncRNA BE503655 in osteosarcoma. We demonstrated for the first time that lncRNA BE503655 as a novel target for osteosarcoma. In the future, exploring the lncRNA BE503655 will provide new evidences for clinical treatment.

## Abbreviations

CCK8: Cell Counting Kit-8
FILNC1: FoxO-induced long non-coding RNA 1
GAPDH: Glyceraldehyde phosphatedehydrogenase
HRP: Horseradish peroxidase
lncRNA: long non-coding RNA
qRT-PCR: Quantitative real time polymerase chain reaction
RIPA: Radio-Immunoprecipitation Assay
RNA-seq: RNA-sequencing
TSLNC8: Tumor suppressive lncRNA on Chromosome 8p12
